# Supplementary material for: DDX1 vesicles control calcium-dependent mitochondrial activity in mouse embryos
Source: Nat Commun. 2022 Jul 1;13:3794. doi: 10.1038/s41467-022-31497-9 (PMC9249788; doi:10.1038/s41467-022-31497-9)
Supplement: Supplementary file 1 — Supplementary Information [file 41467_2022_31497_MOESM1_ESM.pdf]

SUPPLEMENTARY INFORMATION

DDX1 Vesicles Control Calcium-dependent Mitochondrial Activity in Embryos

Yixiong Wang, Lubna Yasmin, Lei Li, Pinzhang Gao, Xia Xu, Xuejun Sun, Roseline Godbout

Corresponding author: Roseline Godbout: rgodbout@ualberta.ca

Supplementary Fig. 1

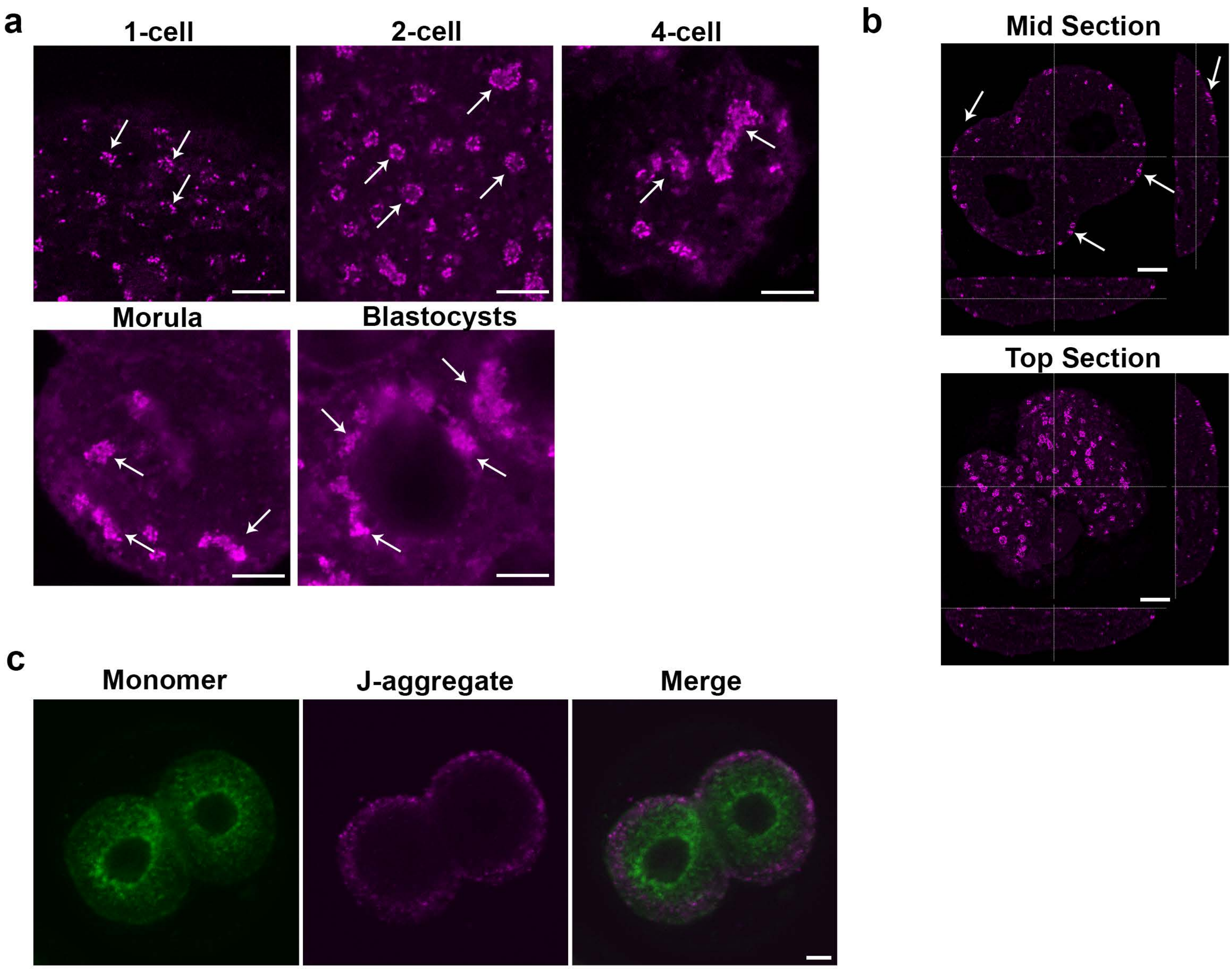

Supplementary Fig. 1. Subplasmalemmal cytoplasmic distribution of DDX1 aggregates and mitochondria with high membrane potential (ΔΨm) in embryos. (**a**) STED microscopy using Atto 550 conjugated anti-DDX1 antibody shows that DDX1 aggregates form ring-like structures at the 2-cell stage. These structures become larger, denser and less ring-like with embryonic development. DDX1 aggregates are mostly observed around the nucleus at the blastocyst stage. A minimum of 2 wild-type crosses were used for each stage (~8 embryos obtained from each cross) and similar results were obtained for all embryos. Scale bars = 5 µm. Arrows point to a subset of DDX1 aggregates. (**b**) DDX1 aggregates are mostly found at the subplasmalemmal cytoplasm in 2-cell embryos. Scale bars = 10 µm. Four wild-type crosses were used for each stage (~8 embryos from each cross). Similar results were obtained for all embryos. Arrows point to DDX1 aggregates at the subplasmalemmal cytoplasm. Images in (**a**) and (**b**) were taken with a Leica SP8 confocal microscope with STED (**a**) and without STED (**b**). (**c**) JC-1 forms J-aggregates at the subplasmalemmal cytoplasm (magenta), and monomers mostly at the inner cytoplasm (green). Three wild-type crosses were used for JC-1 analysis (~8 embryos from each cross). Similar results were obtained for all embryos. Scale bar = 10 µm. Images in (**c**) were taken with an LSM710 confocal microscope.

Supplementary Fig. 2

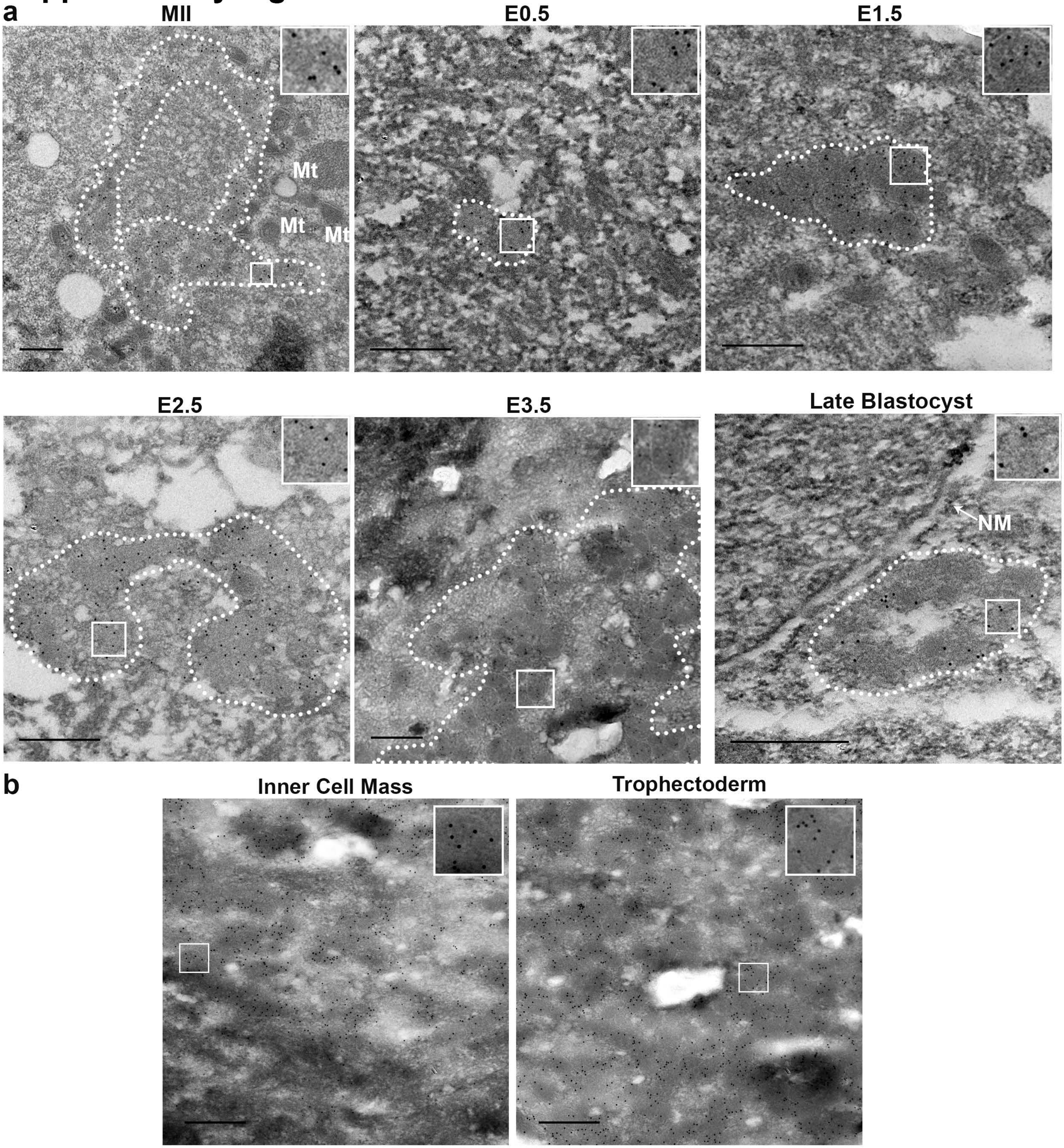

Supplementary Fig. 2. TEM images of DDX1 in MII oocytes and embryos at different stages. (**a**) Membrane bound DDX1-containing vesicles are first visualized in 1-cell embryos, but can no longer be detected at the E4.5 stage when aggregates of DDX1 are mainly observed surrounding the nucleus. DDX1 aggregates (MII stage and blastocyst) and MARVs (E0.5 to E3.5) are indicated by the dotted lines. NM: Nuclear membrane. Mt: Mitochondria. Four embryos from 2 wild-type crosses were used for each stage. Similar results were obtained for all embryos at the same stage. (**b**) Membrane bound DDX1-containing vesicles have a similar appearance in inner cell mass and trophectoderm cells of E3.5 embryos. Scale bars = 0.5 μm. Four embryos from 2 wild-type crosses were used for the analysis. Inner cell mass and trophectoderm cells from the same 4 embryos were used for comparison.

Supplementary Fig. 3

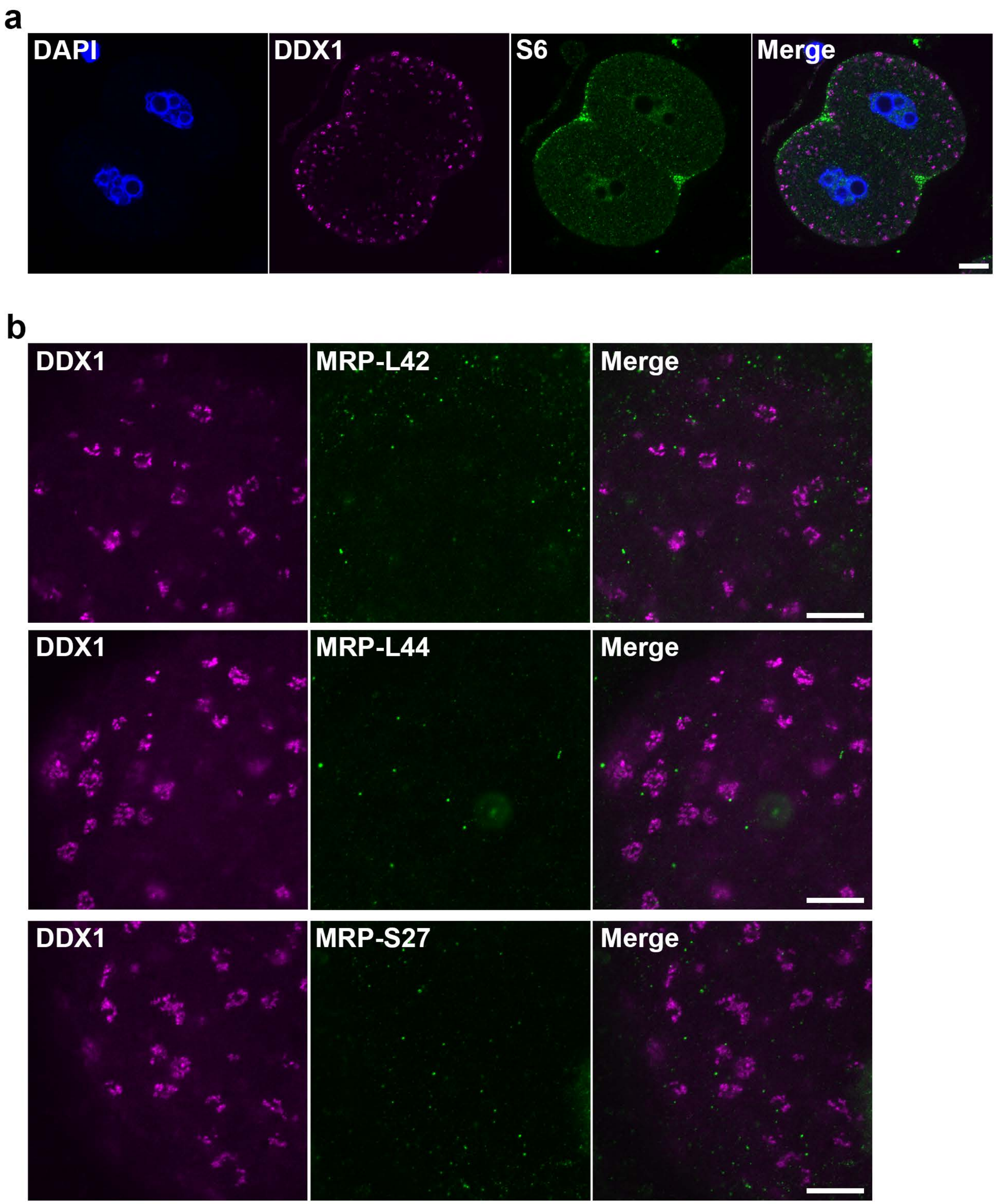

Supplementary Fig. 3. DDX1 does not co-immunostain with ribosomal proteins. (**a**) Cytoplasmic ribosomal protein RPS6 does not co-compartmentalize with DDX1 in 2-cell embryos. Three wild-type crosses were used with similar results obtained for all embryos. Scale bar = 10 µm. (**b**) DDX1 does not co-compartmentalize with mitochondrial ribosomal proteins MRP-L42, MRP-L44 or MRP-S27. Two wild-type crosses were used for each mitochondrial ribosome antibody and similar results were obtained for all embryos. Images were taken using a Leica SP8 STED microscope. Scale bars = 5 µm.

Supplementary Fig. 4

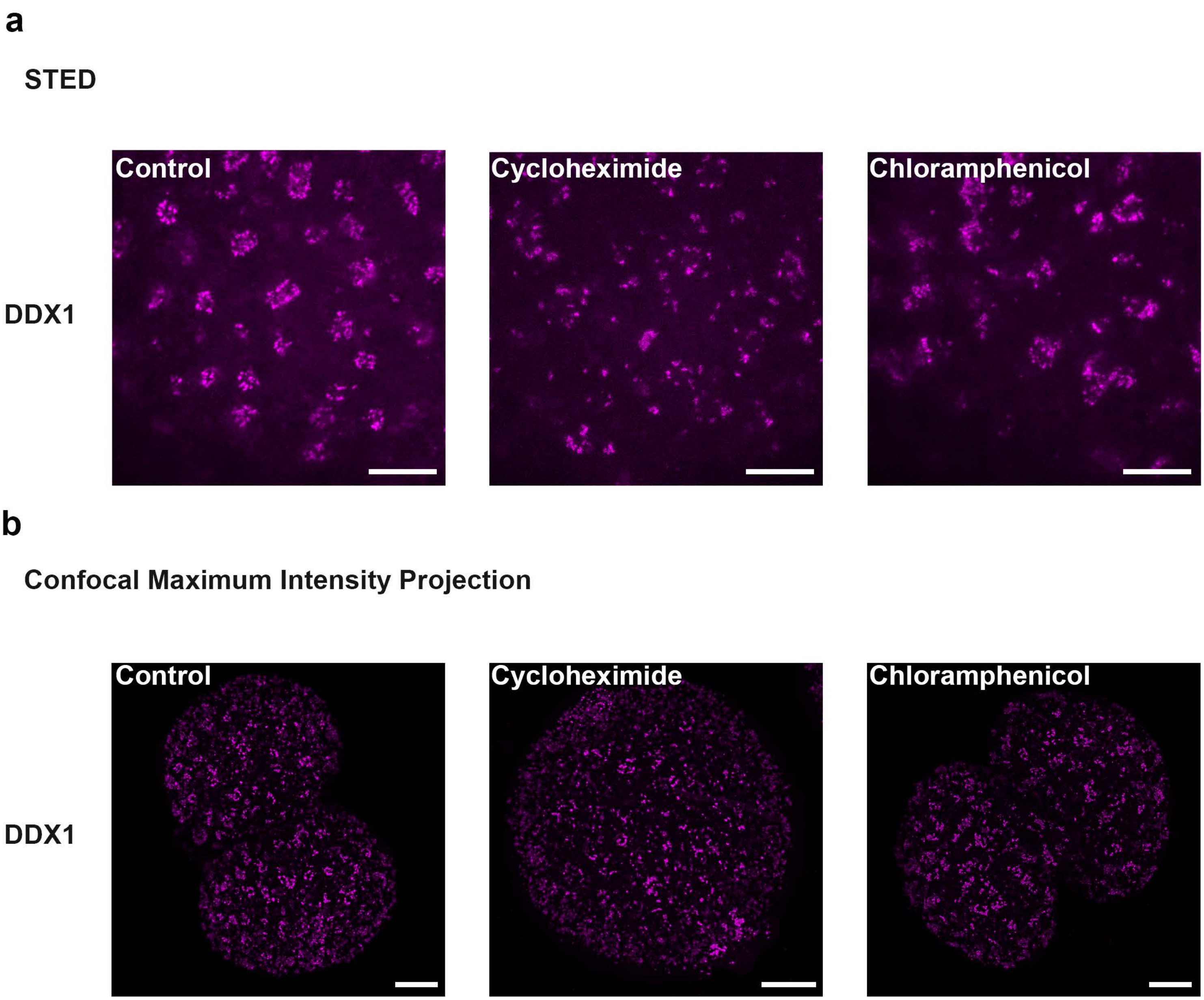

Supplementary Fig. 4. MARVs are disrupted when treated with cycloheximide but not chloramphenicol. (**a**) DDX1 ring-like structures are disrupted when 1-cell embryos are treated with cycloheximide for 8 h, with no obvious effect observed when embryos are treated with chloramphenicol. Scale bars = 5 µm. (**b**) Z-stack images of embryos treated with cycloheximide and chloramphenicol for 8 h are shown in maximum intensity projection. Three wild-type crosses were collected in total with 8 embryos used for each of the three different treatments. Results for each treatment were similar for all embryos. Note that embryos treated with cycloheximide did not progress to the 2-cell stage. Images were taken with a Leica SP8 microscope. Scale bars = 10 µm.

Supplementary Fig. 5

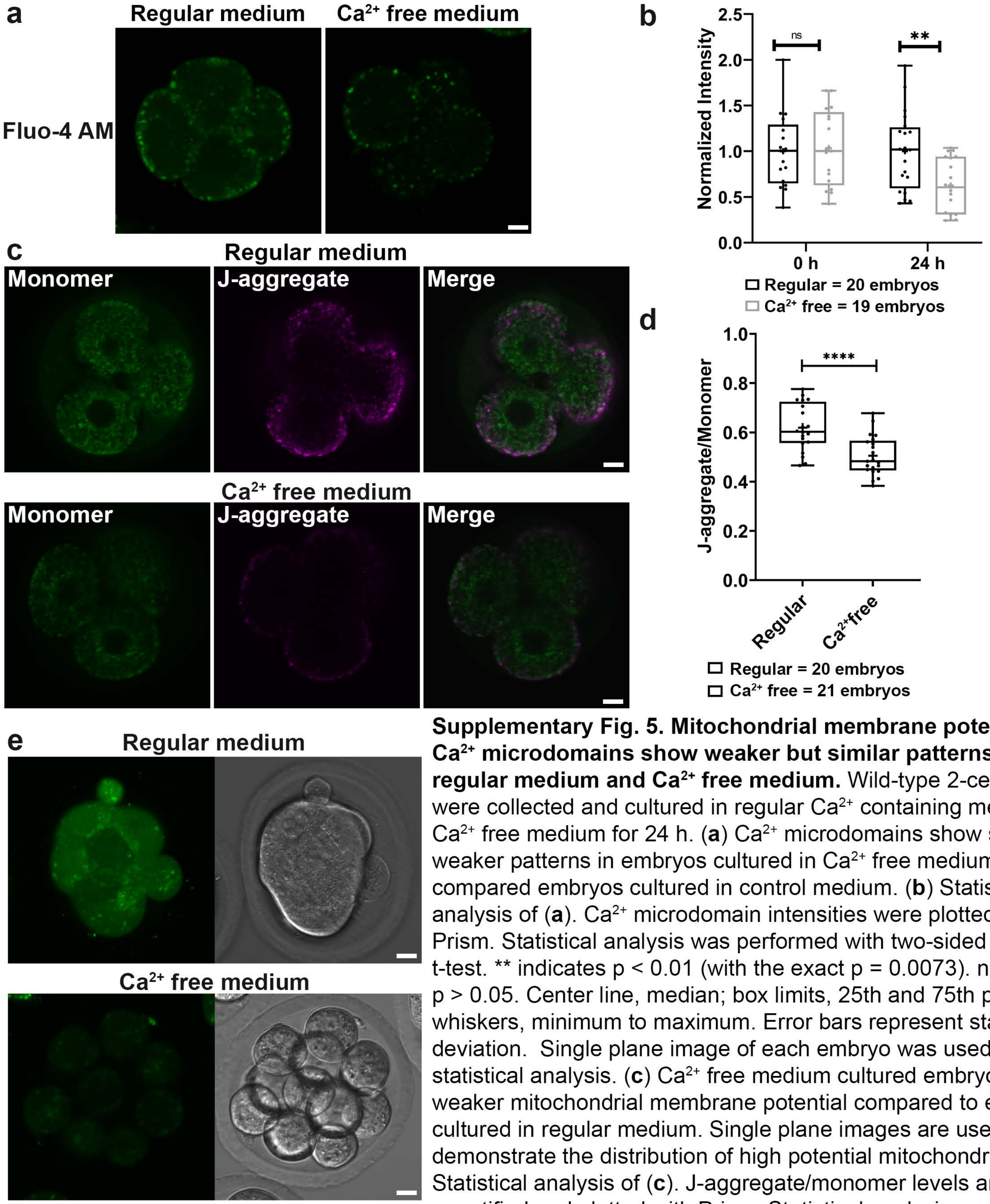

Supplementary Fig. 5. Mitochondrial membrane potential and Ca^{2+} microdomains show weaker but similar patterns in regular medium and Ca^{2+} free medium. Wild-type 2-cell embryos were collected and cultured in regular Ca^{2+} containing medium or Ca^{2+} free medium for 24 h. (**a**) Ca^{2+} microdomains show similar but weaker patterns in embryos cultured in Ca^{2+} free medium compared embryos cultured in control medium. (**b**) Statistical analysis of (**a**). Ca^{2+} microdomain intensities were plotted with Prism. Statistical analysis was performed with two-sided Students' t-test. ** indicates $p < 0.01$ (with the exact $p = 0.0073$). ns indicates $p > 0.05$. Center line, median; box limits, 25th and 75th percentiles; whiskers, minimum to maximum. Error bars represent standard deviation. Single plane image of each embryo was used for the statistical analysis. (**c**) Ca^{2+} free medium cultured embryos showed weaker mitochondrial membrane potential compared to embryos cultured in regular medium. Single plane images are used to better demonstrate the distribution of high potential mitochondria. (**d**) Statistical analysis of (**c**). J-aggregate/monomer levels are quantified and plotted with Prism. Statistical analysis was performed with two-sided Students' t-test. **** indicates $p < 0.0001$ (with the exact $p = 1.67E-05$). Center line, median; box limits, 25th and 75th percentiles; whiskers, minimum to maximum. Error bars represent standard deviation. Z-stack images of each embryo was used for statistical analysis. (**e**) Embryos failed to compact when cultured in Ca^{2+} free medium for 48 h. Embryos from three wild-type crosses showed similar results. Scale bars = 10 μm.

Supplementary Fig. 6

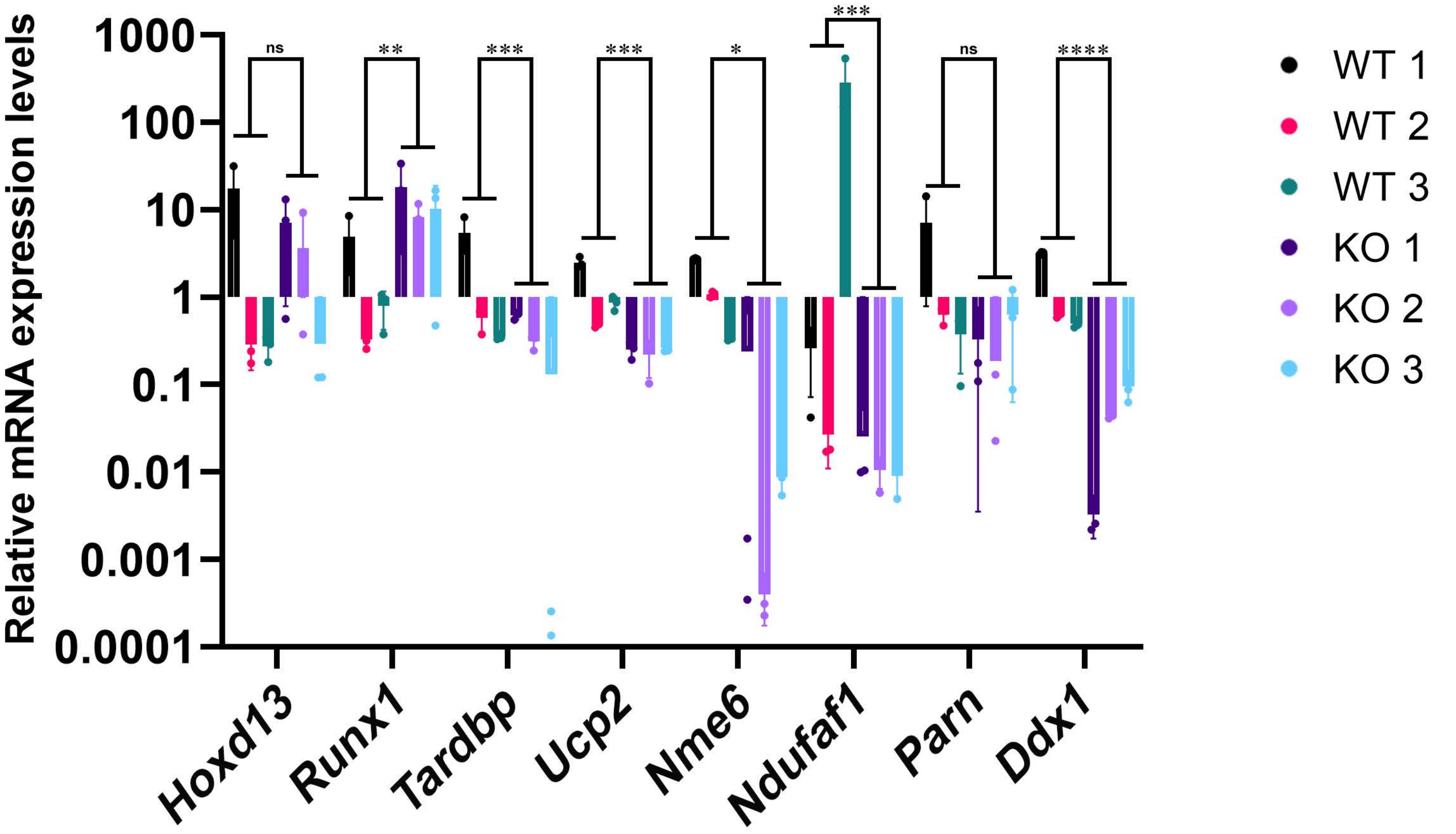

Supplementary Fig. 6. Embryos with low levels of *Ddx1* show altered gene expression compared to wild-type embryos. Seven genes from single embryo RNA sequencing data (Supplementary Dataset 2) were validated by RT-qPCR. Low *Ddx1* embryos (presumed *Ddx1*$^{-/-}$ embryos) have higher levels of *Hoxd13* and *Runx1* mRNAs, and lower levels of *Tardbp, Ucp2, Nme5, Ndufaf1,* and *Parn* mRNAs compared to wild-type embryos. Data are plotted in log10 scale with Prism and statistical significance was calculated with two-sided multiple t-test using the mean value of 3 embryos tested in each group. ns indicates $p > 0.05$; * indicates $p < 0.05$; ** indicates $p < 0.01$; *** indicates $p < 0.001$; **** indicates $p < 0.0001$. The exact p values are 0.41 for *Hoxd13*, 0.0054 for *Runx1*, 0.00077 for *Tardbp* , 0.00039 for *Ucp2*, 0.032 for *Nme6*, 0.0001 for *Ndufaf1*, 0.054 for *Parn*, and 0.000021for *Ddx1*. Error bars represent standard deviation of the data.

Supplementary Fig. 7

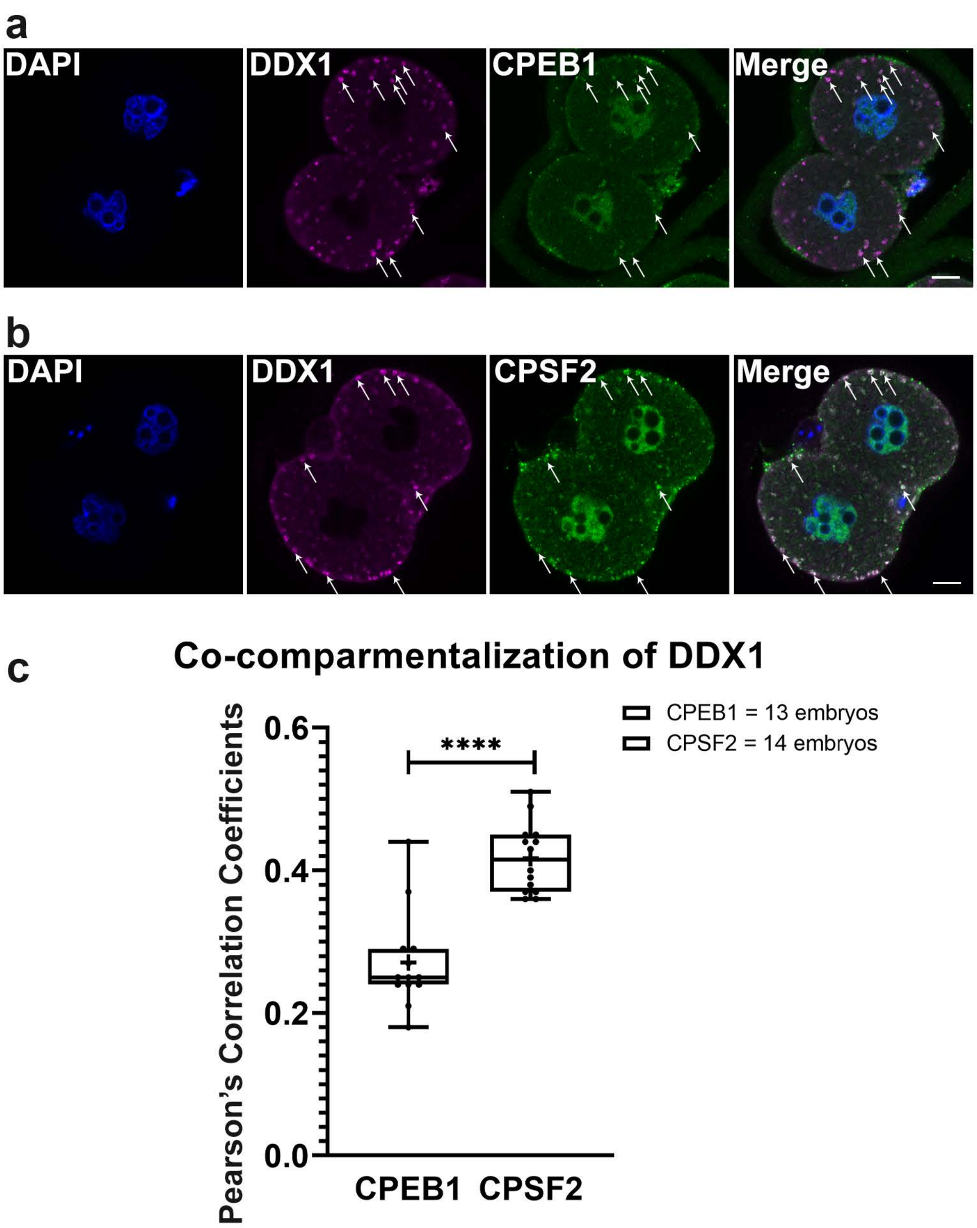

Supplementary Fig. 7. CPEB1 and CPSF2 localize with DDX1 in MARVs. (**a**) DDX1 and CPEB1 co-compartmentalize in 2-cell embryos indicating that these two proteins are present in MARVs. Arrows point to areas of co-compartmentalization. (**b**) DDX1 and CPSF2 co-compartmentalize in 2-cell embryos indicating that these two proteins are present in MARVs. Arrows point to areas of co-compartmentalization. (**c**) Statistical analysis of (**a**) and (**b**). Center line, median; box limits, 25th and 75th percentiles; whiskers, minimum to maximum. Error bars represent standard deviation. Statistical analysis was performed with two-sided Students' t-test. **** indicates $p < 0.0001$. The exact p value is 8.71E-07. Scale bars = 10 μm. Single plane image of each embryo was used for statistical analysis.
