# Supplementary material for: DDX1 vesicles control calcium-dependent mitochondrial activity in mouse embryos
Source: Nat Commun. 2022 Jul 1;13:3794. doi: 10.1038/s41467-022-31497-9 (PMC9249788; doi:10.1038/s41467-022-31497-9)
Supplement: Supplementary file 3 — Description of additional Supplementary File [file 41467_2022_31497_MOESM3_ESM.pdf]

Descriptions of Additional Supplementary Data files

Supplementary Dataset 1. Single embryo mRNA sequencing results.

Supplementary Dataset 2. Primers used for RT-qPCR
