# Supplementary material for: DDX1 vesicles control calcium-dependent mitochondrial activity in mouse embryos
Source: Nat Commun. 2022 Jul 1;13:3794. doi: 10.1038/s41467-022-31497-9 (PMC9249788; doi:10.1038/s41467-022-31497-9)
Supplement: Supplementary file 6 — Reporting Summary [file 41467_2022_31497_MOESM6_ESM.pdf]

nature portfolio

Corresponding author(s): Roseline Godbout

Last updated by author(s): Jun 7, 2022

Reporting Summary

Nature Portfolio wishes to improve the reproducibility of the work that we publish. This form provides structure for consistency and transparency in reporting. For further information on Nature Portfolio policies, see our Editorial Policies and the Editorial Policy Checklist.

Statistics

For all statistical analyses, confirm that the following items are present in the figure legend, table legend, main text, or Methods section.

n/a	Confirmed	
[ ]	[x]	The exact sample size (*n*) for each experimental group/condition, given as a discrete number and unit of measurement
[ ]	[x]	A statement on whether measurements were taken from distinct samples or whether the same sample was measured repeatedly
[ ]	[x]	The statistical test(s) used AND whether they are one- or two-sided<br>*Only common tests should be described solely by name; describe more complex techniques in the Methods section.*
[x]	[ ]	A description of all covariates tested
[ ]	[x]	A description of any assumptions or corrections, such as tests of normality and adjustment for multiple comparisons
[ ]	[x]	A full description of the statistical parameters including central tendency (e.g. means) or other basic estimates (e.g. regression coefficient) AND variation (e.g. standard deviation) or associated estimates of uncertainty (e.g. confidence intervals)
[ ]	[x]	For null hypothesis testing, the test statistic (e.g. *F*, *t*, *r*) with confidence intervals, effect sizes, degrees of freedom and *P* value noted<br>*Give P values as exact values whenever suitable.*
[x]	[ ]	For Bayesian analysis, information on the choice of priors and Markov chain Monte Carlo settings
[x]	[ ]	For hierarchical and complex designs, identification of the appropriate level for tests and full reporting of outcomes
[ ]	[x]	Estimates of effect sizes (e.g. Cohen's *d*, Pearson's *r*), indicating how they were calculated

Our web collection on statistics for biologists contains articles on many of the points above.

Software and code

Policy information about availability of computer code

Data collection	Zeiss software was used for Confocal image acquisition (ZEN version 2011); Leica Application Suite X software was used for STED image acquisition (LAS X version 3.5); Gatan Microscope Suite Software was used for TEM image acquisition (Digital Micrograph, Version 3.23)
Data analysis	ImageJ 1.52e; Coloc2 3.0.5; CLIJx 0.32.1.1; Prism 8; R version 4.0.3; DESeq2 3.15;

For manuscripts utilizing custom algorithms or software that are central to the research but not yet described in published literature, software must be made available to editors and reviewers. We strongly encourage code deposition in a community repository (e.g. GitHub). See the Nature Portfolio guidelines for submitting code & software for further information.

Data

Policy information about availability of data

All manuscripts must include a data availability statement. This statement should provide the following information, where applicable:

- Accession codes, unique identifiers, or web links for publicly available datasets
- A description of any restrictions on data availability
- For clinical datasets or third party data, please ensure that the statement adheres to our policy

All data needed to reproduce and evaluate the conclusions in this paper are present in the main text and supplementary data. Original data of all figures presented in the main text and supplementary data are provided in the source data file. Sequencing data can be found in SRR17058908, SRR17058907, SRR17058906, SRR17058905, SRR17058904, SRR17058903.

The authors declare that all the data supporting the findings of this work are available with the article, its supplementary data files and source data. RNA sequencing data are publicly available under SRR17058908, SRR17058907, SRR17058906, SRR17058905, SRR17058904, SRR17058903.

Human research participants

Policy information about studies involving human research participants and Sex and Gender in Research.

Reporting on sex and gender	N/A
Population characteristics	N/A
Recruitment	N/A
Ethics oversight	N/A

Note that full information on the approval of the study protocol must also be provided in the manuscript.

Field-specific reporting

Please select the one below that is the best fit for your research. If you are not sure, read the appropriate sections before making your selection.

[x] Life sciences [ ] Behavioural & social sciences [ ] Ecological, evolutionary & environmental sciences

For a reference copy of the document with all sections, see nature.com/documents/nr-reporting-summary-flat.pdf

Life sciences study design

All studies must disclose on these points even when the disclosure is negative.

Sample size	The embryo samples were generated from 3-5 independent crosses for statistical analyses as regular molecular biology experiments. Each embryo represents one data point as different embryos represent different individuals.
Data exclusions	No Samples were excluded from the analysis
Replication	All attempts at replication were successful. Each data point represents a single embryo. Crosses were performed a minimum of 2 times for wildtype crosses and 3-5 times for heterozygous crosses.
Randomization	Sample allocation was random
Blinding	The intensity analyses and particle size analyses were calculated blindly to obtain the values for each sample. These values were then used for statistical analysis.

Reporting for specific materials, systems and methods

We require information from authors about some types of materials, experimental systems and methods used in many studies. Here, indicate whether each material, system or method listed is relevant to your study. If you are not sure if a list item applies to your research, read the appropriate section before selecting a response.

Materials & experimental systems

n/a	Involved in the study	
[ ]	[x]	Antibodies
[x]	[ ]	Eukaryotic cell lines
[x]	[ ]	Palaeontology and archaeology
[ ]	[x]	Animals and other organisms
[x]	[ ]	Clinical data
[x]	[ ]	Dual use research of concern

Methods

n/a	Involved in the study	
[x]	[ ]	ChIP-seq
[x]	[ ]	Flow cytometry
[x]	[ ]	MRI-based neuroimaging

Antibodies

Antibodies used	1. Rabbit anti-DDX1 antibody (prepared in-house) and Atto 550-conjugated rabbit anti-DDX1 antibody (conjugation was with the Atto 550 Protein Labeling Kit from Sigma-Aldrich (#51146);<br>2. Mouse anti-RPS6 (Santa Cruz, Cat #sc-13007);

	3. Mouse anti-MRPS27 (Santa Cruz, Cat #sc-390396);<br>4. Mouse anti-MRPL42 (Santa Cruz, Cat #sc-515820);<br>5. Mouse anti-MRPL44 (Santa Cruz, Cat #sc-515503);<br>6. Mouse anti-CPEB1 (Santa Cruz, Cat #sc-514688);<br>7. Goat anti-CPSF2 (Santa Cruz, Cat #sc-26658);<br>8. Alexa Fluor® 647-conjugated donkey anti-mouse IgG (H+L) secondary antibody (Molecular Probes, Thermo Fisher, Cat #A-31571);<br>9. Alexa Fluor® 555-conjugated donkey anti-rabbit IgG (H+L) secondary antibody (Molecular Probes, Thermo Fisher, Cat #A-31572);<br>10. Alexa Fluor® 647-conjugated donkey anti-goat IgG (H+L) secondary antibody (Molecular Probes, Thermo Fisher, Cat #A-21447);<br>11. Goat anti-rabbit 10nm nano-gold (Electron Microscopy Sciences, Cat #25109)
Validation	1. Affinity-purified rabbit anti DDX1 antibody (prepared in-house); Described and validated in our previous paper (Bleoo et al. 2001 DOI: 10.1091/mbc.12.10.3046);<br>2.Atto 550-conjugated affinity-purified rabbit primary FABP7 antibody (conjugation was with the Atto 550 Protein Labeling Kit from Sigma-Aldrich (#51146);<br>3. Mouse anti-RPS6 antibody has been validated according to the manufacturer's website. It has been validated with Xenopus IF, IP and human IF, IP and mouse cell line western blot.<br>4. Anti-MRP-S27 Antibody (A-10) is recommended for detection of MRP-S27 of mouse, rat and human origin by WB, IP, IF, IHC(P) and ELISA<br>5. Anti-MRP-L42 Antibody (H-8) is recommended for detection of MRP-L42 of mouse, rat and human origin by WB, IP, IF and ELISA<br>6. Anti-MRP-L44 Antibody (G-12) is recommended for detection of MRP-L44 of mouse, rat and human origin by WB, IP, IF and ELISA<br>7. Anti-CPEB Antibody (G-6) recommended for detection of CPEB long and short isoforms of mouse, rat and human origin by WB, IP, IF and ELISA<br>8. Anti-CPSF2 Antibody has been used by our lab and published by Li et al. Dynamic Nature of Cleavage Bodies and Their Spatial Relationship to DDX1 Bodies, Cajal Bodies, and Gems. MBoC Mar 2006. doi: 10.1091/mbc.E05-08-0768.<br>9. Goat anti-rabbit 10nm nano-gold antibody has also been used by Anastasia et al. Mitochondria-rough-ER contacts in the liver regulate systemic lipid homeostasis. Cell Reports Mar 2021. doi: 10.1016/j.celrep.2021.108873

Animals and other research organisms

Policy information about studies involving animals; ARRIVE guidelines recommended for reporting animal research, and Sex and Gender in Research

Laboratory animals	FVB/N strain male and female adult mice (8-10 weeks old) were housed in specific pathogen-free conditions with 14 hours light (7am - 9pm) and 10 hours dark (9pm - 7am) cycles at temperature of 20-22 degree Celsius with humidity of 50-60%. Approximately 135 female mice were used for the paper.
Wild animals	The current study does not involve wild animals.
Reporting on sex	Both male and female mice have been used in our current experiments.
Field-collected samples	The current study does not involve any samples collected from the field.
Ethics oversight	All animal experiments were carried out in accordance with the approved guidelines of the Cross Cancer Institute Animal Care Committee (protocol #AC20253).

Note that full information on the approval of the study protocol must also be provided in the manuscript.
